# Nuclear envelope structural defects cause chromosomal numerical instability and aneuploidy in ovarian cancer

**DOI:** 10.1186/1741-7015-9-28

**Published:** 2011-03-26

**Authors:** Callinice D Capo-chichi, Kathy Q Cai, Fiona Simpkins, Parvin Ganjei-Azar, Andrew K Godwin, Xiang-Xi Xu

**Affiliations:** 1 Sylvester Comprehensive Cancer Center, University of Miami Miller School of Medicine, Miami, FL 33136, USA; 2 Department of Medicine, University of Miami Miller School of Medicine, Miami, FL 33136, USA; 3 Ovarian Cancer Program, Fox Chase Cancer Center, Philadelphia, PA 19111, USA; 4 Department of Obstetrics and Gynecology, University of Miami Miller School of Medicine, Miami, FL 33136, USA; 5 Department of Pathology, University of Miami Miller School of Medicine, Miami, FL 33136, USA; 6 Department of Pathology and Laboratory Medicine, University of Kansas Medical Center, Kansas City, KS 66160, USA

## Abstract

**Background:**

Despite our substantial understanding of molecular mechanisms and gene mutations involved in cancer, the technical approaches for diagnosis and prognosis of cancer are limited. In routine clinical diagnosis of cancer, the procedure is very basic: nuclear morphology is used as a common assessment of the degree of malignancy, and hence acts as a prognostic and predictive indicator of the disease. Furthermore, though the atypical nuclear morphology of cancer cells is believed to be a consequence of oncogenic signaling, the molecular basis remains unclear. Another common characteristic of human cancer is aneuploidy, but the causes and its role in carcinogenesis are not well established.

**Methods:**

We investigated the expression of the nuclear envelope proteins lamin A/C in ovarian cancer by immunohistochemistry and studied the consequence of lamin A/C suppression using siRNA in primary human ovarian surface epithelial cells in culture. We used immunofluorescence microscopy to analyze nuclear morphology, flow cytometry to analyze cellular DNA content, and fluorescence *in situ *hybridization to examine cell ploidy of the lamin A/C-suppressed cells.

**Results:**

We found that nuclear lamina proteins lamin A/C are often absent (47%) in ovarian cancer cells and tissues. Even in lamin A/C-positive ovarian cancer, the expression is heterogeneous within the population of tumor cells. In most cancer cell lines, a significant fraction of the lamin A/C-negative population was observed to intermix with the lamin A/C-positive cells. Down regulation of lamin A/C in non-cancerous primary ovarian surface epithelial cells led to morphological deformation and development of aneuploidy. The aneuploid cells became growth retarded due to a p53-dependent induction of the cell cycle inhibitor p21.

**Conclusions:**

We conclude that the loss of nuclear envelope structural proteins, such as lamin A/C, may underlie two of the hallmarks of cancer - aberrations in nuclear morphology and aneuploidy.

## Background

A deformed and enlarged nuclear morphology is a common characteristic of cancer cells, and the "roundness" of the nucleus is a good indicator to distinguish benign, low grade, and malignant cells [1,2]. In the clinical setting, the morphology of the nucleus is used universally for diagnostic and prognostic prediction of malignancies of tumor cells, referred to as "nuclear grade" [1,2]. The most well known diagnostic test based on cell and nuclear morphology is the cervical Papanicolaou (PAP) smear test [3]. In PAP smears, cells collected from a swab of the cervix are examined under microscope to determine the presence of large and atypical nuclei, which serves as an initial diagnosis of cervical or uterine cancer. The simple procedure was invented in the 1930s, widely implemented by the 1960s, still universally practiced worldwide today, and is credited for saving millions of lives.

In the last 5 decades, much research has been devoted to understand the molecular basis for the atypical and enlarged nucleus that accompanies malignancy. In ovarian cancer, nuclear size and morphology correlate with the degree of genetic changes and can be used to distinguish low- from high-grade serous cancer, as well as to predict outcome [4-6]. Molecular changes in the nuclear matrix and/or nuclear envelope have been postulated, and deformation of nuclear morphology was shown to associate with oncogenic signaling [7-9], but no definite conclusions have been reached regarding the molecular basis of nuclear deformation in malignant cells [1,10].

Another hallmark of cancer cells, first recognized over one hundred years ago by Boveri [11,12] is aneuploidy, or an abnormal and unbalanced number of chromosomes compared to diploid normal cells. The majority (around 90%) of human ovarian cancers are aneuploid and possess a hyperdiploid (> 46) to subtetraploid (< 96) chromosome number http://www.ncbi.nlm.nih.gov/sky/skyweb.cgi?form_type=submitters. Cancer cells within one tumor or cell line are not uniform in chromosomal number, indicating the presence of chromosomal numerical instability in cancer cells [13]. Although an association between aneuploidy and malignancy has been well recognized, the causes and significance of aneuploidy in cancer remain unsettled [14-17]. Formation of aneuploid cells was found to be an early event in the development of ovarian cancer, suggesting aneuploidy contributed to cancer initiation [18]. Mitotic failure, tetraploid intermediates, and subsequent unbalanced cytokineses are the most common cause of aneuploidy [19-21]. Frequently, tetraploid cells are the products of mitotic regression after failed cytokinesis and the intermediates that produce aneuploid cells in subsequent mitotic events [20]. Nevertheless, in ovarian cancer, the molecular basis for the causes of aneuploidy is largely unknown.

In mammalian cells, loss or mutation of nuclear envelope structural proteins such as emerin [22] or lamin A/C [23] produces nuclear morphological deformation. Lamin A and lamin C are differentially spliced forms of the same gene and differ only at the C-terminal domain [24]. Lamin A/C expression is absent in embryonic stem cells and early embryos, and is progressively expressed in nearly all tissues at later developmental stages [25]. The initiation of lamin A/C expression is associated with cell differentiation, which suggests that lamin A/C expression may serve as a limit on the plasticity of cells for further developmental events [25,26]. Additionally, the cell types that seem to lack lamin A/C, such as embryonic carcinoma cells and some cells of the spleen, thymus, bone marrow and intestine in the adult mouse may fall into the "stem cell" category, but the correlation will need to be carefully tested [25,26]. Loss or mutation of nuclear envelope proteins such as lamin A/C [27] or emerin [28] causes muscular dystrophy, severe premature aging (progeria) [29], and several additional diseases catalogued as laminopathies [30], but a link between the nuclear envelope protein deficiency with the deformed nuclear morphology in cancer cells has not been established.

Loss of lamin A/C expression is often found in cancer cells [26,31], including leukemia and lymphoma [32,33], colon cancer [34,35], prostatic cancer [9], lung cancer [36], and gastric cancer [37]. One study reported the identification of high lamin A/C expression in a fraction of ovarian cancer cases using high-density protein microarrays [38]. However, normal ovarian surface epithelial cells were also found to be high in lamin A/C staining [38]. The observation may be explained by the low fraction of epithelial cells relative to the total ovarian tissue masses used for protein array analysis.

Recently, we found that the nuclear envelope protein emerin is lost in a fraction of ovarian cancer cells [39]. Here, we further explored the expression of additional nuclear envelope proteins in ovarian cancer and studied the phenotypes for the loss of lamin A/C in ovarian epithelial cells.

## Materials and methods

### Tumor specimens and immunohistochemistry

The current study utilized archived tumor tissues and cell lines prepared from human ovaries obtained from prophylactic surgeries. The tissues and cells lines were obtained from Fox Chase Cancer Center tumor bank. These tissues are designed for research only and contain no link to personal information of the donors. The study was reviewed by the Institutional Review Board (IRB) of both Fox Chase Cancer Center and the University of Miami Miller School of Medicine, and was approved and judged as non-human subject research.

The three ovarian tumor tissue microarrays (duplicate core of 120 tumor tissues and 5 controls) and 20 prophylactic oophorectomies were provided by the Tumor Bank of Fox Chase Cancer Center. These ovarian tissues and tumor specimens were described in more detail previously [40,41]. Immunostaining was performed using the mouse DAKO Envision TM^+ ^System and the Peroxidase (DAB) Kit (Dako Carpinteria, CA) as previously described [24-26]. Negative controls were performed by replacing the primary antibodies with non-immunized IgG.

### Human ovarian surface epithelial and cancer cell cultures

Human ovarian surface epithelial (HOSE) cells were established from ovaries obtained from prophylactic oophorectomies [41]. The donors were usually individuals who were either BRCA1/2 carriers or had a family history of breast or ovarian cancer but there was no indication of cancer at the time of surgery. Each fresh specimen of intact whole ovary from surgeries was immersed in medium and the ovarian surface was gently scraped with a rubber policeman to collect cells. The ovarian tissues were then subjected to histology analysis by pathologists to confirm the absence of microscopic tumors. HOSE cells were transfected with SV40 T-antigen to prepare human "immortalized" ovarian (HIO) cells. These HIO cells have a longer lifespan in culture and can be cultured for 20 to 50 passages before undergoing senescence, compared to HOSE cells that can only be maintained for 5 to 7 passages [42,43].

Ovarian epithelial cancer cell lines were cultured in (D)MEM with 10% FBS as previously reported [42,43]. The OVCAR lines were previously established by Thomas Hamilton [42] and the others (A1847, A2780, ES2, and SKOV-3) were obtained from American Type Culture Collection.

### Probes for Northern blot and antibodies for Western blot

Probes for lamin A/C, emerin, and lamin B1 were obtained by reverse transcriptase-polymerase chain reaction (PCR) using total RNA extracts from HOSE cells at passage 2 as a template. The ascension number, sense primer (SP), and anti-sense primer (ASP) are as following: lamin B1 (BC078178): cggtg tacat cgaca aggtg (SP), cagct gttgc tgcat ttgat (ASP); BAF (BC005942); gaacc gttac gggaa ctgaa (SP), tcaaa cggat tgaaa gtgag g (ASP); and lamin A/C (NM_005572): tctgc tgaga ggaac agcaa (SP), ggtga tggag caggt catct (ASP). The PCR products were sequenced to confirm the identity.

Mouse anti-emerin, rabbit anti-p21, and goat anti-lamin B antibodies were purchased from Santa Cruz Biotechnology Inc (Santa Cruz, CA). Anti-p53 mouse IgGs were purchased from Dako (Carpinteria, CA) and mouse anti-beta-tubulin from Sigma St. Louis, MO.

### Small interfering RNA (shRNA) transfection

siRNA oligos and shRNA plasmids directed against human lamin A/C were purchased from Santa Cruz Biotechnology and were transfected into cells using Lipofectamine 2000 (Invitrogen, Carlsbad, CA) according to the manufacturer's protocol. Cells were retrieved 72 hours after transfection for analysis by Western blot, Northern blot, or immunofluorescence microscopy.

### Flow cytometry analysis

For sorting of live cells, 0.5 μM vybrant violet dye (Molecular Probe) was added and incubated for 30 minutes at 37°C. For fixed cell analysis, the trypsinized cells were resuspended in ice-cold ethanol/PBS (70% v/v) and kept at -20°C until ready to use. Before flow cytometry analysis, the fixed cells were resuspended in 0.5 μM vybrant violet dye or propidium iodide and RNase A solution. Cell sorting and flow analyses were performed on a LSR Fortessa driven by BD's FACS Diva software, version 6.1.3 (Becton Dickinson, San Jose, CA).

### Immunofluorescence microscopy

Cells were fixed with 4% paraformaldehyde, permeabilized with 0.5% Triton X-100 for 5 minutes, blocked with 3% BSA in PBS containing 0.1% Tween-20 for 30 minutes, incubated for 1 hour at 37°C with primary antibodies, and subsequently with secondary antibodies conjugated with AlexaFluor 488 (green fluorescence) or AlexaFluor 594 (red fluorescence) (Molecular Probes/Invitrogen, Carlsbad, CA). The nuclei were stained with Hoechst 33342 (Molecular Probes). Cells were mounted/sealed in anti-fade reagent containing 0.1 M n-propyl gallate (pH 7.4) and 90% glycerol in PBS. A Zeiss microscope and AxioCam Camera and AxioVision software 4.8 were used for image acquisition.

### Cytogenetic analysis

Chromosome number counting and fluorescence in situ hybridization (FISH) analysis were performed by the University of Miami Cytogenetic Core Facility. Briefly, the cells were exposed to colcemid (demecolcine) (0.015 mg/ml) for 12 hours, resuspended in 10 ml hypotonic buffer (75 mM KCl) for 20 minutes, and then kept in 1 ml of chilled fixative (methanol/acetic acid, 3/1). The cell suspension was dropped on wet slides and heated (70°C) overnight, dyed with Wright stain, and analyzed to count 50 metaphases for each sample in duplicate. For FISH analysis, an alpha-satellite DNA probe for chromosome X centromeres (Xp11.1-q11.1) and a probe for chromosome Y satalite III (Yq12) were used in interphase cells. The pre-labeled FISH probes were purchased from Abbott Molecular (Abbott Park, Illinois, USA).

## Results

### Lamin A/C expression is frequently lost in ovarian cancer cells and tissues

**Figure 1:**
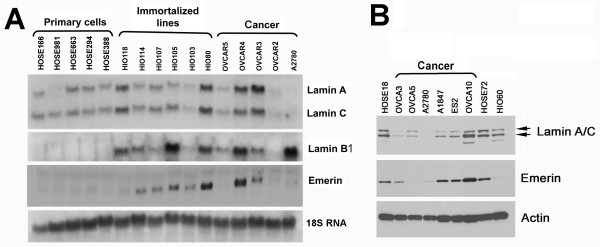
**Expression of nuclear envelope proteins in ovarian epithelial and cancer cell lines**. Primary non-cancer cells and cancer cell lines were analyzed for mRNA and protein expression of nuclear envelope proteins lamin A/C, lamin B1, and emerin. (**A**) Total RNA was isolated and analyzed by Northern blot of primary human ovarian surface epithelial cells (HOSE166, HOSE981, HOSE663, HOSE294, and HOSE388), immortalized lines (HIO118, HIO114, HIO107, HIO105, HIO80), and ovarian cancer cells (OVCAR8, OVCAR4, OVCAR3, OVCAR5, and A2780) for the expression of lamin A/C, lamin B1, and emerin. (**B**) Total cell lysates from two lines of primary ovarian surface epithelial cells (HOSE18, HOSE72), an immortalized line (HIO60), and six ovarian cancer lines (OVCAR3, OVCAR5, OVCAR10, A2780, A1847, and ES2), were prepared from cultures for Western blot analysis.

Since the nuclear envelope structural protein emerin was found lost in a fraction (38%) of ovarian cancer [39], we examined if the expression of other nuclear envelope proteins might account for nuclear morphological deformation of emerin-expressing ovarian cancer cells. First, the expression of several nuclear envelope proteins was investigated by Northern blot in a panel of ovarian surface epithelial cells and cancer cell lines (Figure 1A). mRNA for lamin B1 and emerin was found to be generally higher in ovarian cancer and immortalized (HIO) cells than primary human ovarian surface epithelial (HOSE) cells (Figure 1A). Lamin A/C mRNA is expressed in some but is reduced in other (OVCAR2 and A2780) ovarian cancer cells. We noticed that lamin A but not lamin C mRNA is missing in HOSE981 cells, though information is not available for us to correlate with potential disease or phenotype of the individual. For proteins, emerin is often missing or reduced in ovarian cancer lines as reported previously [39], and noticeably, lamin A/C proteins are generally reduced in ovarian cancer cells (Figure 1B). It appears that emerin mRNA but not protein level is often increased in "immortalized" ovarian epithelial and ovarian cancer cells, and the expression of emerin mRNA and protein levels do not correlate. Thus, we conclude that in ovarian cancer cells emerin is lost/reduced at the protein but not the mRNA level; and lamin A and lamin C are reduced both at the mRNA and protein levels.

**Figure 2:**
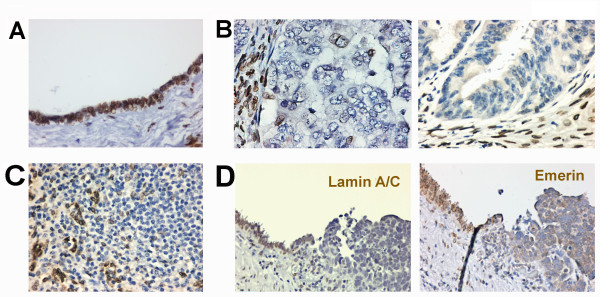
**Loss of lamin A/C expression in ovarian cancers**. Ovarian normal and tumor tissues were analyzed by immunostaining for lamin A/C. (**A**) A representative example shows that lamin A/C is strongly stained around the nucleus of normal ovarian surface epithelial cells. The nuclei are generally smooth and oval-shaped in all normal ovarian surface epithelial cells. (**B**) Two examples show loss of lamin A/C expression in ovarian carcinomas. Some stromal cells show strong lamin A/C staining around the nucleus, serving as an internal positive control. The nuclear morphology of tumor cells is heterogeneous, and some cells with large and deformed nuclei are present. (**C**) A representative example of ovarian carcinomas showing heterogeneous expression of lamin A/C among the tumor cells. (**D**) An example of ovarian carcinomas contiguous with the benign ovarian surface epithelium. Lamin A/C is lost in the transformed cells. In an adjacent section, the loss of lamin A/C correlates with the distribution of emerin to the cytoplasm of tumor cells.

Next, we examined human ovarian tumors for lamin A/C expression by immunohistochemistry. Normal ovarian tissues show strong and uniform lamin A/C staining around the nuclear envelope of all ovarian surface epithelial cells (Figure 2A). Out of 108 informative ovarian carcinomas [see Additional file 1: Table S1], we found that 51 (47%) lack lamin A/C expression in epithelial tumor cells, while on the same field of the slide some stromal cells are positive, as shown in two examples (Figure 2B). Additionally, the majority of ovarian carcinomas that were catalogued as lamin A/C positive, contain a fraction of lamin A/C-negative cells intermingling with lamin A/C-positive cells [see Additional file 1: Table S1], as shown by an example (Figure 2C). Another case is shown for an area containing both normal ovarian surface epithelial cells and neoplastic cells, which shows that the loss of lamin A/C correlates closely with neoplastic transformation and an increase in nuclear size (Figure 2D). Also, emerin staining in the same area from an adjacent section becomes cytoplasmic instead of at the nuclear envelope, accompanying the loss of lamin A/C (Figure 2E). The correlation between loss of lamin A/C expression and emerin cellular distribution is consistent with the fact that lamin A is essential for nuclear envelope localization of emerin [44]. We conclude that the loss or abnormal distribution of lamin A/C occurs in a significant percentage (47%) of human ovarian cancers, and the rest show a variable degree of loss of lamin A/C expression in a fraction of tumor cells [see Additional file 1: Table S1]. Thus, in addition to the previous reports for leukemia [32] and B-cell lymphomas [33], colon cancer [34,35], prostatic cancer [9], lung cancer [36], and gastric cancer [37], lamin A/C is also often lost or reduced in ovarian cancer.

### Most ovarian cancer cells and tissues have a heterogeneous Lamin A/C protein expression pattern

Furthermore, the tumors grouped as lamin A/C-positive usually also contain a fraction of lamin A/C-negative cells, ranging from 10% to 40% [see Additional file 1: Table S1]. In contrast, all ovarian epithelial cells in normal tissues have strong lamin A/C staining around the nuclear envelope (Figure 2A). Thus, nearly all ovarian cancer cases have some degrees of nuclear envelope structural defect due to the loss of lamin A/C (reported here) or emerin [39] protein in a fraction to all of the tumor cell population.

**Figure 3:**
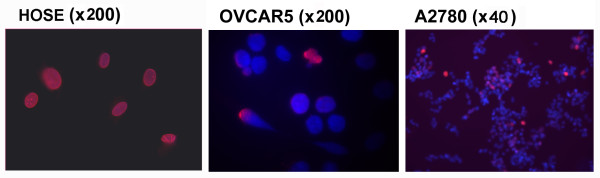
**Heterogeneous lamin A/C expression in ovarian cancer cells**. Lamin A/C expression in ovarian normal and cancer epithelial cells was analyzed by immunofluorescence microscopy for DAPI (blue) and lamin A/C (red). Primary ovarian surface epithelial (HOSE) cells exhibit a uniform staining pattern for lamin A/C. All ovarian cancer cell lines show heterogeneous staining of lamin A/C within the cell population, as shown in examples, the OVCAR5 and A2780 ovarian cancer cells.

Using immunofluorescence microscopy, we examined the presence of lamin A/C in individual cells in culture. In primary HOSE cells, all the cells (100%) stained strongly for lamin A/C, showing a smooth and oval-shaped nuclear morphology (Figure 3A). In all lines of cancer cells examined, lamin A/C is lost in some lines, or the expression is heterogeneous in the cell population. For example, in OVCAR5 and A2780 ovarian cancer cells, lamin A/C is detectable but weak by Western blot (Figure 1B); however, most of the cells are negative and only a small fraction of positive cells are present, as detected by immunofluorescence microscopy (Figure 3).

### Lamin A/C suppression in human ovarian epithelial cells causes nuclear morphological deformation

**Figure 4:**
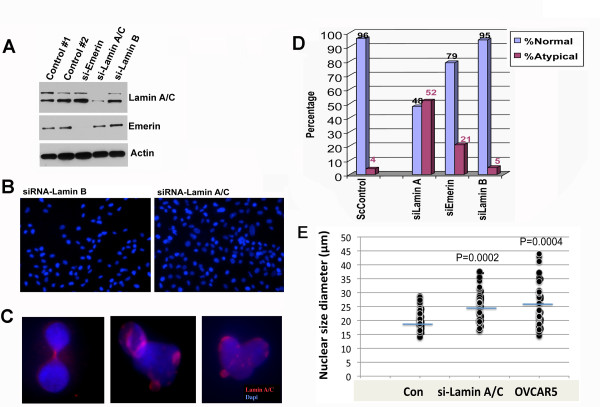
**Suppression of lamin A/C expression in ovarian surface epithelial cells leads to a deformed nuclear shape**. Primary HOSE cells were treated with siRNA to lamin A/C for three days and were analyzed for the effect on nuclear size and morphology. (**A**) Western blot shows the specific suppression of protein by siRNA to emerin, lamin A/C, and lamin B1 in primary HOSE cells. (**B**) A low magnification image (× 200) shows the presence of a high percentage of cells with large and deformed nuclear morphology following suppression of lamin A/C by siRNA in primary HOSE cells for three days. Suppression of lamin B had no significant effect on nuclear morphology. (**C**) Examples shown at high magnification (× 1,000) of individual deformed nuclei with reduced and patchy lamin A/C staining. (**D**) The nuclear morphological abnormality was scored by computer-assisted image analysis. About 100 nuclei in each group treated by siRNA were analyzed and the percentage of normal and atypical nuclear morphology is shown. ScControl: scrambled controls. (**E**) The nuclear size/diameter was scored by computer-assisted image analysis (AxioVision software 4) following shRNA treatment for 3 days. Nuclear sizes were plotted following computer-assisted imaging analysis of 50 nuclei randomly selected from each group, compared to OVCAR5 ovarian cancer cells.

To explore the potential role of the loss of lamin A/C in ovarian cancer, we investigated the consequence of lamin A/C suppression in HOSE cells. Suppression of lamin A/C proteins with siRNA oligonucleotides was achieved as indicated by Western blot analysis (Figure 4A). We noticed a dramatic occurrence of cells with large and irregularly shaped nuclei two to three days after targeting lamin A/C, but not lamin B, with siRNA (Figure 4B). Nuclei with chromatin bridges, conjoined, and heart-shape appearance are common in lamin A/C-suppressed cells (Figure 4C). In several experiments using multiple HOSE cell preparations, lamin A/C down regulation consistently resulted in around 50% of the cells having large and atypical nuclear morphology. Conversely, cells transfected with control siRNA exhibited mostly smooth, round or oval shaped nuclei with less than 4% of cells having atypical nuclei. The extent of lamin A/C down regulation on nuclear size and morphology was quantified by computer-assisted imaging analysis, and the results indicated that lamin A/C down regulation caused a drastic change in nuclear shape (Figure 4D). In comparison, down regulation of emerin caused nuclear morphological deformation in around 20% of cells, consistent with our previous report [39].

The nuclear sizes of the cell population also changed upon lamin A/C suppression (Figure 4E), as determined using computer software (AxioVision software 4). The diameter of the nucleus had a range of 13 to 29 μm in HOSE cells and shifted up to 16 to 38 μm in the lamin A/C-suppressed cells (Figure 4E). The average size increased from 18 to 29 μm upon lamin A/C suppression, and the change was found statistically significant (*P *< 0.005). Following lamin A/C suppression in HOSE cells, the nuclear size distribution of the cell population resembles OVCAR5 ovarian cancer cells (Figure 4E). The variation in nuclear size may be caused by chromatin organization, condensation, and DNA content. OVCAR5 has 51 to 57 chromosomes per cell http://www.ncbi.nlm.nih.gov/sky/skyweb.cgi?form_type=submitters[13]. Thus, the experiments showed that the loss of lamin A/C leads to atypical nuclear morphology and increased nuclear size in HOSE cells, resembling those of cancer cells. Similar to OVCAR5 cells, the increased nuclear size distribution in lamin A/C-suppressed HOSE cells was speculated to be caused by increased DNA content and changes in chromatin organization. These experiments have been repeated three times over a one-year period using three independent preparations of HOSE cells, and the reproducibility of the results was very good.

### Suppression of Lamin A/C leads to polyploidy and aneuploidy in ovarian surface epithelial cells

**Figure 5:**
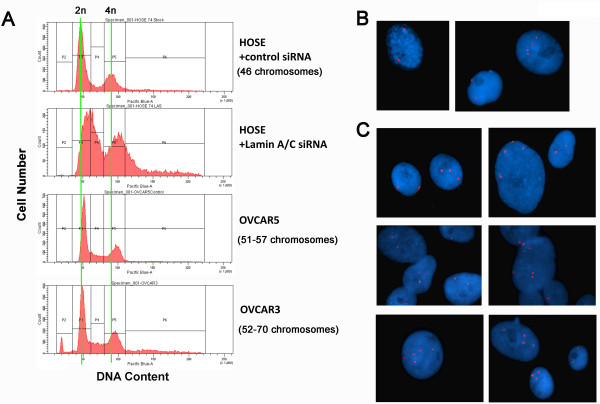
**Lamin A/C suppression in primary ovarian epithelial cells results in increased nuclear size, aneuploidy, and polyploidy**. Primary HOSE cells were transfected with control or shRNA to suppress lamin A/C and were subjected to analysis for DNA content and ploidy. (**A**) The shRNA-treated and control HOSE cells were sorted by flow cytometry to compare DNA content. Two aneuploid ovarian cancer cell lines, OVCAR5 (51 to 57 chromosomes per cell) and OVCAR3 (52 to 70 chromosomes per cell) were analyzed for comparison. A subtle increase in DNA content was observed in lamin A/C-suppressed HOSE cells. (**B**) *In situ *hybridization with X chromosome centromere probe of control HOSE cells treated with scrambled shRNA. Two representative fields are shown. (**C**) *In situ *hybridization with X centromere probe of lamin A/C-suppressed HOSE cells. Six representative fields are shown for the presence of 3, 4, and other multiple X-chromosome-containing nuclei.

Next, we analyzed cellular DNA content in HOSE cells following lamin A/C suppression. First, we determined the DNA content of the cell populations by flow cytometry (Figure 5A). Lamin A/C-suppressed HOSE cells exhibited a slight but recognizable shift in DNA content compared to HOSE cells of both 2n and 4n populations (Figure 5A). The subtle increases in cellular DNA content upon lamin A/C suppression were comparable to those of OVCAR5 (51 to 57 chromosomes per cell) and OVCAR3 (52 to 70 chromosomes per cell) cells, performed in the flow cytometry as controls (Figure 5A). Thus, we conclude that the lamin A/C-suppressed cells achieved aneuploidy and a chromosomal distribution similar to the aneuploid ovarian cancer cells (OVCAR5 and OVCAR3).

These cells were further analyzed by FISH using an X centromere probe. Typically, the control cells showed two X-chromosome markers per cell, indicating a diploid ploidy (Figure 5B). Rare (less than one per 100 nucleus examined) tetraploid cells were observed, as shown in an example (Figure 5B). In contrast, three to four X-chromosomes per nucleus were detected in 37% of the lamin A/C-suppressed HOSE cells, as shown in examples from six images (Figure 5C). Thus, suppression of lamin A/C leads to the formation of tetraploid and aneuploid ovarian epithelial cells. The wide distribution of nuclear sizes and the broad range of DNA contents in the lamin A/C-suppressed cells is consistent with a variation of chromosomal numbers and aneuploidy of the cells; however, we were not able to determine the exact chromosomal number in each cell due to difficulty in obtaining a sufficient number of mitotic cells (discussed below).

### Suppression of Lamin A/C leads to reduced mitosis and retarded cell growth

**Figure 6:**
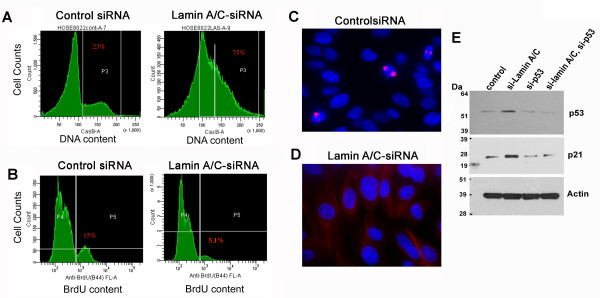
**Lamin A/C suppression in primary ovarian epithelial cells results in growth retardation**. Primary HOSE cells were transfected with control or shRNA to suppress lamin A/C expression. The cells were analyzed for DNA content and bromodeoxyuridine (BrdU) incorporation simultaneously by flow cytometry. (**A**) Primary HOSE cells were analyzed by flow cytometry for DNA content following treatment with control or lamin A/C shRNA for three days. Lamin A/C-suppressed cells have > 2n DNA in 75% of cells, compared to 23% of control cells. (**B**) The same cell populations were incubated with BrdU for one hour in culture, and then fixed and stained with FITC-anti-BrdU prior to flow cytometry to determine BrdU incorporation as an indicator of proliferation. (**C**) The control cells show a high percentage of mitotic nuclei as shown by immunofluorescence microscopy of beta-tubulin staining of the mitotic spindles. (**D**) In comparison, beta-tubulin-immunostained mitotic spindles are rare in lamin A/C-suppressed cells. (**E**) The HOSE cells were treated with siRNA-p53 and/or siRNA-lamin A/C for three days and then analyzed for p53 and p21 by Western blot.

In several attempts to analyze the exact chromosome number by cytogenetic approach, we failed to collect a sufficient number of mitotic nuclei in lamin A/C-suppressed cells, while control cells readily formed metaphase spreads. This observation suggests that suppression of lamin A/C may interfere with cell proliferation and normal mitosis. We performed flow cytometry to analyze simultaneously DNA content (Figure 6A) and bromodeoxyuridine (BrdU) incorporation as an indicator of DNA synthesis (Figure 6B) in the same cell population. Indeed, in lamin A/C-suppressed HOSE cells, 75% of the cells have DNA content higher than 2n, but only 5.1% of the cells had high BrdU incorporation. In comparison, 23% of the control cells had DNA content higher than 2n, and 15% of the cells had high BrdU uptake. These results indicate that the lamin A/C-suppressed cells were aneuploid or polyploid, and were not in S-phase or actively undergoing replication.

Although the primary HOSE cells proliferated slowly in culture (compared to cancer cells), we observed that about 12% of the control cells in culture were undergoing mitosis using beta-tubulin staining to mark the mitotic spindles (Figure 6C). In contrast, mitotic spindles were rare (< 1%) in lamin A/C-suppressed cells (Figure 6D), indicating a reduced cell division following the loss of lamin A/C and aneuploidy. It is known that the p53/p21 pathway is activated to mediate growth arrest of aneuploid cells [45]. Indeed, we found that p53 and p21 proteins were increased in lamin A/C-suppressed, aneuploid cells (Figure 6E). The induced cell cycle inhibitor p21 following lamin A/C-suppression is p53-dependent, since simultaneous suppression of both lamin A/C and p53 did not stimulate an increased p21 expression. It was reasoned that the lamin A/C-suppressed and aneuploid cells might overcome growth arrest once p53 function is lost, and future experiments are to determine the long-term consequence of lamin A/C suppression in the absence of p53.

## Discussion

A significant understanding of ovarian cancer biology and genetics has been achieved [46,47], though the causes and importance of chromosomal instability as well as nuclear atypia are still topics of research. We found that lamin A/C is absent in a significant fraction of ovarian (47%) cancers. Intriguingly, most ovarian carcinoma tissues and cancer cell lines exhibit a heterogeneous pattern of lamin A/C expression in the population of cancer cells, which may account for the variation of nuclear shapes within an ovarian tumor. Lamin A/C expression is regulated during cell differentiation and embryonic development, but the expression is not known to be regulated in a cell cycle-dependent manner [25,26]. Thus the heterogeneous lamin A/C expression in cancer cells may link to heterogeneity of tumor cells.

Despite its being such a prominent feature of malignancy, what role a deformed nucleus may play in the development of cancer is unclear. The identification of the loss of nuclear envelope structural proteins emerin and lamin A/C as the molecular basis of nuclear deformation in cancer cells may provide some explanation. We found that suppression of emerin [39] or lamin A/C (current study) lead to mitotic failure and the formation of polypoid and subsequent aneuploid cells. The essential role of lamin A/C in mitosis and the formation of the envelope to house the newly formed nuclei in dividing cells have been shown in *Caenorhabditis elegans *[48,49], which has only one lamin gene. Lamin suppression results in the formation of chromatin bridges, large and deformed nuclei, and ultimately developmental failure [49]. Emerin suppression only slightly affects cell division and its role is redundant with MAN gene in *C. elegans *[50].

In mammals, neither lamin A/C [51] nor emerin [52,53] is essential for cell division and development as shown by gene knockout in mice. However, lamin A/C-deficient cells exhibit a phenotype of chromosomal numerical instability, though the mechanism was not analyzed [54]. Mutations in the lamin A/C gene have also been shown to affect cell cycle and mitosis in human cells [55,56]. We also report an increased mitotic failure and the formation of polyploidy and aneuploidy in HOSE cells when emerin expression is suppressed [39]. In the current study, we conclude that though lamin A/C is not essential for the initial division of ovarian epithelial cells in culture, nevertheless, the loss of lamin A/C increases mitotic failure and the development of polyploidy and subsequent aneuploidy. We further speculate that nuclear structural defects as a consequence of the loss of lamin A/C (and also loss of emerin) may be a principal mechanism for the chromosomal numerical instability, and the underlying cause of aneuploidy in ovarian cancer. Since lamin A/C expression has been found to be lost in several cancer types [31], loss of lamin A/C expression may be the possible common cause of nuclear atypia and chromosomal numerical instability observed in many cancer types.

A characteristic of laminopathies (disorders caused by mutated and defective lamin A/C) is genomic instability [30,54], which is shared by cancer. However, laminopathies are not linked to a predisposition to cancer. In fact, we observed that suppression of lamin A/C results in a lowered rate of mitosis and cell growth, coincident with polyploidy and aneuploidy. We reasoned that loss of lamin A/C and subsequent aneuploidy by itself is not oncogenic, but actually reduces the efficiency in cell division and proliferation. Aneuploidy is not tolerated in development, has severe consequences on cell proliferation, and can be detrimental to individual normal cells [57,58]. However, with additional oncogenic changes (such as p53 mutation) to bypass cell growth regulation and cellular stress, chromosomal instability brought by the loss of nuclear structural integrity may enable the cells to undergo oncogenic evolution and development of malignant tumors [59,60]. Thus, neoplastic cells may acquire a nuclear structural defect and trade cell growth efficiency for versatility and agility in chromosomal instability. Indeed, ovarian cancer accumulates vast amounts of seemingly random genomic changes [13,61], indicative of chromosomal numerical instability. Both p53 mutation and acquisition of aneuploidy are closely associated early events [18,62], and p53 mutation is common in high-grade ovarian cancer [46,47,63,64], allowing the survival and proliferation of aneuploid cells.

Aneuploidy activates the p53/p21 pathway [45]. Indeed we found that p53 and p21 are increased in lamin A/C-suppressed cells, presumably mediating the observed cell growth arrest. In our experiments, unfortunately, the siRNA-suppression of p53 is short-lived, not permitting our study to determine if loss of both of p53 and lamin A/C might allow survival of aneuploid cells and the selection of clones with a chromosomal composition presenting a neoplastic phenotype. Proper experimental systems (mutant mice) are currently under development to test this hypothesis.

## Conclusions

In summary, we report the finding of the frequent loss and aberrant expression of lamin A/C in ovarian cancer. The experimental results support that a nuclear envelope structural defect caused by the loss of lamin A/C may be the molecular basis of two of the prominent hallmarks of human cancer, nuclear morphological deformation and aneuploidy.

## Competing interests

The authors declare that they have no competing interests.

## Authors' contributions

CDC performed the majority of the experiments included in this manuscript, including cell culture study of expression by Western blot, flow cytometry, immunofluorescence microscopy, and cell growth assay. KQC performed immunohistochemistry of ovarian tumor tissue microarray, and participated in reading and photographing of the slides. FS participated in cell culture and reviewing and interpretation of data. PGA reviewed the ovarian carcinoma histology. AKG provided primary HOSE cells, tumor tissues, and performed Northern blot analysis. CDC prepared the first draft of the manuscript, and all authors were involved in editing and writing. CDC and XXX first made the initial observation and conceived the basic hypothesis of the study. All authors participated in discussion and refining of the ideas and design of the experiments.

## Funding Supports

These studies were supported by funds from concept awards BC097189 and BC076832 from Department of Defense (USA). Grants R01 CA095071, R01 CA099471, and CA79716 to X.X. Xu from NCI, NIH also contributed to the studies. The early stage of this work was also supported by Ovarian Cancer SPORE P50 CA83638 (PI: RF Ozols).

## Pre-publication history

The pre-publication history for this paper can be accessed here:

http://www.biomedcentral.com/1741-7015/9/28/prepub

## Supplementary Material

Additional file 1 **Table S1**. TMA of ovarian carcinomas stained with lamin A/C Click here for file
