# Supplementary material for: Nuclear envelope structural defects cause chromosomal numerical instability and aneuploidy in ovarian cancer
Source: BMC Med. 2011 Mar 26;9:28. doi: 10.1186/1741-7015-9-28 (PMC3072346; doi:10.1186/1741-7015-9-28)
Supplement: Additional file 1 — Table S1. TMA of ovarian carcinomas stained with lamin A/C [file 1741-7015-9-28-S1.DOCX]

**Table S1.** **TMA of ovarian carcinomas stained with lamin A/C**

| **TMA** | **Row** | **Column** | **REGISTRY_HISTOLOGY** | **REGISTRY_GRADE** | **PATH**  **STAGE** | **Lamin A/C staining** | | |
| --- | --- | --- | --- | --- | --- | --- | --- | --- |
|  |  |  |  |  |  | **Normal** | **Tumor** | |
|  |  |  |  |  |  | **intensity** | **intensity** | **% positive** |
| 09-16 | C | 2 | high-grade serous carcinoma | POORLY DIFFERENTIATED | 2A |  | 2 | 70% |
| 09-16 | C | 3 | high-grade serous carcinoma | POORLY DIFFERENTIATED | 2A |  | 2 | 70% |
| 09-16 | C | 4 | high-grade serous carcinoma | MODERATELY DIFFERENTIATED | 3B | 2 | 0 |  |
| 09-16 | C | 5 | high-grade serous carcinoma | MODERATELY DIFFERENTIATED | 3B |  | 2 | 10% |
| 09-16 | C | 6 | high-grade serous carcinoma | NOT DETERMINED; N/A; UNK PRIMARIES, ETC. | X |  | 2 | 80% |
| 09-16 | C | 7 | Papillary serous cystadenocarcinoma | NOT DETERMINED; N/A; UNK PRIMARIES, ETC. | X |  | 2 | 70% |
| 09-16 | C | 8 | Papillary adenocarcinoma, NOS | POORLY DIFFERENTIATED | X |  | / |  |
| 09-16 | C | 9 | Papillary adenocarcinoma, NOS | POORLY DIFFERENTIATED | X |  | 2 | 70% |
| 09-16 | C | 10 | high-grade serous carcinoma | POORLY DIFFERENTIATED | 3C |  | 1 | 30% |
| 09-16 | C | 11 | high-grade serous carcinoma | POORLY DIFFERENTIATED | 3C |  | 1 | 20% |
| 09-16 | C | 12 | high-grade serous carcinoma | MODERATELY DIFFERENTIATED | X | 2 | 0 |  |
| 09-16 | C | 13 | high-grade serous carcinoma | MODERATELY DIFFERENTIATED | X | 2 | 0 |  |
| 09-16 | D | 2 | high-grade serous carcinoma | MODERATELY DIFFERENTIATED | 3C | 2 | 2 | 40% |
| 09-16 | D | 3 | high-grade serous carcinoma | MODERATELY DIFFERENTIATED | 3C |  | 2 | 20% |
| 09-16 | D | 4 | high-grade serous carcinoma | NOT DETERMINED; N/A; UNK PRIMARIES, ETC. | 4 | 2 | 1 | 50% |
| 09-16 | D | 5 | high-grade serous carcinoma | NOT DETERMINED; N/A; UNK PRIMARIES, ETC. | 4 |  | 1 | 50% |
| 09-16 | D | 6 | high-grade serous carcinoma | POORLY DIFFERENTIATED | 3C |  | 0 |  |
| 09-16 | D | 7 | high-grade serous carcinoma | POORLY DIFFERENTIATED | 3C |  | 1 | 60% |
| 09-16 | D | 8 | high-grade serous carcinoma | UNDIFFERENTIATED | 3C |  | 0 |  |
| 09-16 | D | 9 | high-grade serous carcinoma | UNDIFFERENTIATED | 3C |  | 0 |  |
| 09-16 | D | 10 | high-grade serous carcinoma | UNDIFFERENTIATED | 3C |  | 0 |  |
| 09-16 | D | 11 | high-grade serous carcinoma | UNDIFFERENTIATED | 3C |  | 0 |  |
| 09-16 | D | 12 | high-grade serous carcinoma | POORLY DIFFERENTIATED | 3C | 2 | 0 |  |
| 09-16 | D | 13 | high-grade serous carcinoma | POORLY DIFFERENTIATED | 3C | 2 | 0 |  |
| 09-16 | E | 2 | high-grade serous carcinoma | MODERATELY DIFFERENTIATED | 3C |  | 1 | 40% |
| 09-16 | E | 3 | high-grade serous carcinoma | MODERATELY DIFFERENTIATED | 3C |  | 0 |  |
| 09-16 | E | 4 | Serous surface papillary carcinoma | UNDIFFERENTIATED | 3C |  | 0 |  |
| 09-16 | E | 5 | Serous surface papillary carcinoma | UNDIFFERENTIATED | 3C |  | 0 |  |
| 09-16 | E | 6 | high-grade serous carcinoma | UNDIFFERENTIATED | 3C |  | 1 | 20% |
| 09-16 | E | 7 | high-grade serous carcinoma | UNDIFFERENTIATED | 3C |  | 1 | 20% |
| 09-16 | E | 8 | high-grade serous carcinoma | UNDIFFERENTIATED | X |  | 1 | 30% |
| 09-16 | E | 9 | high-grade serous carcinoma | UNDIFFERENTIATED | X |  | 1 | 10% |
| 09-16 | E | 10 | Mucinous adenocarcinoma | MODERATELY DIFFERENTIATED | 3B |  | 1 | 70% |
| 09-16 | E | 11 | Mucinous adenocarcinoma | MODERATELY DIFFERENTIATED | 3B |  | / |  |
| 09-16 | E | 12 | Serous surface papillary carcinoma | UNDIFFERENTIATED | X |  | 1 | 60% |
| 09-16 | E | 13 | Serous surface papillary carcinoma | UNDIFFERENTIATED | X |  | 1 | 60% |
| 09-16 | F | 2 | high-grade serous carcinoma | POORLY DIFFERENTIATED | 3A |  | 0 |  |
| 09-16 | F | 3 | high-grade serous carcinoma | POORLY DIFFERENTIATED | 3A |  | 0 |  |
| 09-16 | F | 4 | Endometrioid adenocarcinoma, NOS | WELL DIFFERENTIATED | 2B |  | 0 |  |
| 09-16 | F | 5 | Endometrioid adenocarcinoma, NOS | WELL DIFFERENTIATED | 2B |  | 0 |  |
| 09-16 | F | 6 | Endometrioid adenocarcinoma, NOS | UNDIFFERENTIATED | 2A |  | 1 | 40% |
| 09-16 | F | 7 | Endometrioid adenocarcinoma, NOS | UNDIFFERENTIATED | 2A |  | 1 | 40% |
| 09-16 | F | 8 | Serous surface papillary carcinoma | MODERATELY DIFFERENTIATED | 3C |  | 1 | 20% |
| 09-16 | F | 9 | Serous surface papillary carcinoma | MODERATELY DIFFERENTIATED | 3C |  | 1 | 30% |
| 09-16 | F | 10 | high-grade serous carcinoma | POORLY DIFFERENTIATED | X |  | 0 |  |
| 09-16 | F | 11 | high-grade serous carcinoma | POORLY DIFFERENTIATED | X |  | 0 |  |
| 09-16 | F | 12 | Serous surface papillary carcinoma | POORLY DIFFERENTIATED | X |  | 1 | 30% |
| 09-16 | F | 13 | Serous surface papillary carcinoma | POORLY DIFFERENTIATED | X |  | 1 | 30% |
| 09-16 | G | 2 | Serous surface papillary carcinoma | POORLY DIFFERENTIATED | 4 |  | 1 | 80% |
| 09-16 | G | 3 | Serous surface papillary carcinoma | POORLY DIFFERENTIATED | 4 |  | 2 | 80% |
| 09-16 | G | 4 | Serous surface papillary carcinoma | POORLY DIFFERENTIATED | 3C |  | 1 | 60% |
| 09-16 | G | 5 | Serous surface papillary carcinoma | POORLY DIFFERENTIATED | 3C |  | 1 | 40% |
| 09-16 | G | 6 | high-grade serous carcinoma | POORLY DIFFERENTIATED | 3B |  | 0 |  |
| 09-16 | G | 7 | high-grade serous carcinoma | POORLY DIFFERENTIATED | 3B |  | 0 |  |
| 09-16 | G | 8 | Serous surface papillary carcinoma | NOT DETERMINED; N/A; UNK PRIMARIES, ETC. | 3C | 1 | 0 |  |
| 09-16 | G | 9 | Serous surface papillary carcinoma | NOT DETERMINED; N/A; UNK PRIMARIES, ETC. | 3C | 1 | 0 |  |
| 09-16 | G | 10 | Serous surface papillary carcinoma | POORLY DIFFERENTIATED | 1B |  | 0 |  |
| 09-16 | G | 11 | Serous surface papillary carcinoma | POORLY DIFFERENTIATED | 1B |  | 0 |  |
| 09-16 | G | 12 | Serous surface papillary carcinoma | POORLY DIFFERENTIATED | 3C |  | 1 | 60% |
| 09-16 | G | 13 | Serous surface papillary carcinoma | POORLY DIFFERENTIATED | 3C |  | 0 |  |
| 09-17 | C | 2 |  |  |  |  |  |  |
| 09-17 | C | 3 |  |  |  |  |  |  |
| 09-17 | C | 4 | high-grade serous carcinoma | POORLY DIFFERENTIATED | X |  | 2 | 70% |
| 09-17 | C | 5 | high-grade serous carcinoma | POORLY DIFFERENTIATED | X |  | 2 | 70% |
| 09-17 | C | 6 | high-grade serous carcinoma | MODERATELY DIFFERENTIATED | X |  | 2 | 70% |
| 09-17 | C | 7 | high-grade serous carcinoma | MODERATELY DIFFERENTIATED | X |  | 2 | 70% |
| 09-17 | C | 8 | Serous surface papillary carcinoma | UNDIFFERENTIATED | 3C |  | 1 | 30% |
| 09-17 | C | 9 | Serous surface papillary carcinoma | UNDIFFERENTIATED | 3C |  | 1 | 40% |
| 09-17 | C | 10 | high-grade serous carcinoma | UNDIFFERENTIATED | 3C |  | 1 | 60% |
| 09-17 | C | 11 | high-grade serous carcinoma | UNDIFFERENTIATED | 3C |  | 1 | 60% |
| 09-17 | C | 12 | high-grade serous carcinoma | POORLY DIFFERENTIATED | 2C |  | 2 | 80% |
| 09-17 | C | 13 | high-grade serous carcinoma | POORLY DIFFERENTIATED | 2C |  | 2 | 70% |
| 09-17 | D | 2 | Serous cystadenocarcinoma, NOS | MODERATELY DIFFERENTIATED | X |  | 3 | 805 |
| 09-17 | D | 3 | Serous cystadenocarcinoma, NOS | MODERATELY DIFFERENTIATED | X |  | 3 | 80% |
| 09-17 | D | 4 | Serous surface papillary carcinoma | NOT DETERMINED; N/A; UNK PRIMARIES, ETC. | 1A |  | 3 | 80% |
| 09-17 | D | 5 | Serous surface papillary carcinoma | NOT DETERMINED; N/A; UNK PRIMARIES, ETC. | 1A |  | 3 | 80% |
| 09-17 | D | 6 | Serous cystadenocarcinoma, NOS | POORLY DIFFERENTIATED | 3C |  | 1 | 50% |
| 09-17 | D | 7 | Serous cystadenocarcinoma, NOS | POORLY DIFFERENTIATED | 3C |  | 1 | 20% |
| 09-17 | D | 8 | high-grade serous carcinoma | POORLY DIFFERENTIATED | X |  | 3 | 50% |
| 09-17 | D | 9 | high-grade serous carcinoma | POORLY DIFFERENTIATED | X |  | 3 | 60% |
| 09-17 | D | 10 | high-grade serous carcinoma | POORLY DIFFERENTIATED | X |  | 2 | 80% |
| 09-17 | D | 11 | high-grade serous carcinoma | POORLY DIFFERENTIATED | X |  | 2 | 50% |
| 09-17 | D | 12 | Serous cystadenocarcinoma, NOS | UNDIFFERENTIATED | 3C |  | 3 | 90% |
| 09-17 | D | 13 | Serous cystadenocarcinoma, NOS | UNDIFFERENTIATED | 3C |  | 3 | 90% |
| 09-17 | E | 2 | Serous surface papillary carcinoma | NOT DETERMINED; N/A; UNK PRIMARIES, ETC. | 3C |  | 2 | 90% |
| 09-17 | E | 3 | Serous surface papillary carcinoma | NOT DETERMINED; N/A; UNK PRIMARIES, ETC. | 3C |  | 2 | 80% |
| 09-17 | E | 4 | Serous cystadenocarcinoma, NOS | UNDIFFERENTIATED | 3C |  | 1 | 60% |
| 09-17 | E | 5 | Serous cystadenocarcinoma, NOS | UNDIFFERENTIATED | 3C |  | 1 | 60% |
| 09-17 | E | 6 | high-grade serous carcinoma | UNDIFFERENTIATED | 3C |  | 1 | 10% |
| 09-17 | E | 7 | high-grade serous carcinoma | UNDIFFERENTIATED | 3C |  | 1 | 20% |
| 09-17 | E | 8 | Serous surface papillary carcinoma | POORLY DIFFERENTIATED | X | 3 | 0 |  |
| 09-17 | E | 9 | Serous surface papillary carcinoma | POORLY DIFFERENTIATED | X | 3 | / |  |
| 09-17 | E | 10 | Serous cystadenocarcinoma, NOS | UNDIFFERENTIATED | 3B |  | / |  |
| 09-17 | E | 11 | Serous cystadenocarcinoma, NOS | UNDIFFERENTIATED | 3B |  | 1 | 30% |
| 09-17 | E | 12 | Serous surface papillary carcinoma | UNDIFFERENTIATED | 3C |  | / |  |
| 09-17 | E | 13 | Serous surface papillary carcinoma | UNDIFFERENTIATED | 3C |  | / |  |
| 09-17 | F | 2 | high-grade serous carcinoma | POORLY DIFFERENTIATED | X |  | 2 | 80% |
| 09-17 | F | 3 | high-grade serous carcinoma | POORLY DIFFERENTIATED | X |  | 2 | 70% |
| 09-17 | F | 4 | high-grade serous carcinoma | NOT DETERMINED; N/A; UNK PRIMARIES, ETC. | 3C |  | 2 | 90% |
| 09-17 | F | 5 | high-grade serous carcinoma | NOT DETERMINED; N/A; UNK PRIMARIES, ETC. | 3C |  | 2 | 90% |
| 09-17 | F | 6 | Serous surface papillary carcinoma | UNDIFFERENTIATED | 3C |  | 2 | 80% |
| 09-17 | F | 7 | Serous surface papillary carcinoma | UNDIFFERENTIATED | 3C |  | 2 | 80% |
| 09-17 | F | 8 | Serous cystadenocarcinoma, NOS | UNDIFFERENTIATED | 3C |  | 0 |  |
| 09-17 | F | 9 | Serous cystadenocarcinoma, NOS | UNDIFFERENTIATED | 3C |  | 0 |  |
| 09-17 | F | 10 | high-grade serous carcinoma | POORLY DIFFERENTIATED | 3B |  | 0 |  |
| 09-17 | F | 11 | high-grade serous carcinoma | POORLY DIFFERENTIATED | 3B |  | 0 |  |
| 09-17 | F | 12 | high-grade serous carcinoma | UNDIFFERENTIATED | X |  | 2 | 70% |
| 09-17 | F | 13 | high-grade serous carcinoma | UNDIFFERENTIATED | X |  | 2 | 70% |
| 09-17 | G | 2 | high-grade serous carcinoma | POORLY DIFFERENTIATED | 3C |  | 1 | 60% |
| 09-17 | G | 3 | high-grade serous carcinoma | POORLY DIFFERENTIATED | 3C |  | 1 | 20% |
| 09-17 | G | 4 | high-grade serous carcinoma | UNDIFFERENTIATED | 1A |  | 1 | 30% |
| 09-17 | G | 5 | high-grade serous carcinoma | UNDIFFERENTIATED | 1A |  | 1 | 40% |
| 09-17 | G | 6 |  |  |  |  |  |  |
| 09-17 | G | 7 |  |  |  |  |  |  |
| 09-17 | G | 8 | high-grade serous carcinoma | MODERATELY DIFFERENTIATED | 3C |  | 2 | 80% |
| 09-17 | G | 9 | high-grade serous carcinoma | MODERATELY DIFFERENTIATED | 3C |  | 2 | 80% |
| 09-17 | G | 10 |  |  |  |  |  |  |
| 09-17 | G | 11 |  |  |  |  |  |  |
| 09-17 | G | 12 | high-grade serous carcinoma | POORLY DIFFERENTIATED | X |  | 1 | 20% |
| 09-17 | G | 13 | high-grade serous carcinoma | POORLY DIFFERENTIATED | X |  | 0 |  |
| 09-18 | C | 2 | Endometrioid adenocarcinoma, NOS | MODERATELY DIFFERENTIATED | 1A |  | 2 | 40% |
| 09-18 | C | 3 | Endometrioid adenocarcinoma, NOS | MODERATELY DIFFERENTIATED | 1A |  | 2 | 40% |
| 09-18 | C | 4 | Adenocarcinoma, NOS | POORLY DIFFERENTIATED | 3C |  | 2 | 60% |
| 09-18 | C | 5 | Adenocarcinoma, NOS | POORLY DIFFERENTIATED | 3C |  | 2 | 30% |
| 09-18 | C | 6 | Endometrioid adenocarcinoma, NOS | MODERATELY DIFFERENTIATED | 3C |  | 3 | 70% |
| 09-18 | C | 7 | Endometrioid adenocarcinoma, NOS | MODERATELY DIFFERENTIATED | 3C |  | 3 | 70% |
| 09-18 | C | 8 | Endometrioid adenocarcinoma, NOS | NOT DETERMINED; N/A; UNK PRIMARIES, ETC. | 1C | 3 | 0 |  |
| 09-18 | C | 9 | Endometrioid adenocarcinoma, NOS | NOT DETERMINED; N/A; UNK PRIMARIES, ETC. | 1C |  | / |  |
| 09-18 | C | 10 | Endometrioid adenocarcinoma, NOS | WELL DIFFERENTIATED | 3C | 3 | 2 | 80% |
| 09-18 | C | 11 | Endometrioid adenocarcinoma, NOS | WELL DIFFERENTIATED | 3C |  | 2 | 80% |
| 09-18 | C | 12 | Endometrioid adenocarcinoma, NOS | MODERATELY DIFFERENTIATED | X |  | 2 | 60% |
| 09-18 | C | 13 | Endometrioid adenocarcinoma, NOS | MODERATELY DIFFERENTIATED | X |  | / |  |
| 09-18 | D | 2 | Adenocarcinoma, NOS | POORLY DIFFERENTIATED | 3C |  | 2 | 60% |
| 09-18 | D | 3 | Adenocarcinoma, NOS | POORLY DIFFERENTIATED | 3C |  | 1 | 30% |
| 09-18 | D | 4 | Endometrioid adenocarcinoma, NOS | POORLY DIFFERENTIATED | X |  | 2 | 60% |
| 09-18 | D | 5 | Endometrioid adenocarcinoma, NOS | POORLY DIFFERENTIATED | X |  | 2 | 60% |
| 09-18 | D | 6 | Clear cell adenocarcinoma, NOS | POORLY DIFFERENTIATED | X |  | 1 | 40% |
| 09-18 | D | 7 | Clear cell adenocarcinoma, NOS | POORLY DIFFERENTIATED | X |  | 2 | 60% |
| 09-18 | D | 8 | Endometrioid adenocarcinoma, NOS | POORLY DIFFERENTIATED | 1A |  | 0 |  |
| 09-18 | D | 9 | Endometrioid adenocarcinoma, NOS | POORLY DIFFERENTIATED | 1A |  | 0 |  |
| 09-18 | D | 10 | Clear cell adenocarcinoma, NOS | POORLY DIFFERENTIATED | 1C |  | / |  |
| 09-18 | D | 11 | Clear cell adenocarcinoma, NOS | POORLY DIFFERENTIATED | 1C |  | 0 |  |
| 09-18 | D | 12 | Clear cell adenocarcinoma, NOS | NOT DETERMINED; N/A; UNK PRIMARIES, ETC. | X |  | 1 | 60% |
| 09-18 | D | 13 | Clear cell adenocarcinoma, NOS | NOT DETERMINED; N/A; UNK PRIMARIES, ETC. | X |  | 1 | 80% |
| 09-18 | E | 2 | Adenosquamous carcinoma | POORLY DIFFERENTIATED | 4 |  | 3 | 90% |
| 09-18 | E | 3 | Adenosquamous carcinoma | POORLY DIFFERENTIATED | 4 |  | 3 | 90% |
| 09-18 | E | 4 | Mucinous cystadenocarcinoma, NOS | WELL DIFFERENTIATED | X |  | 2 | 60% |
| 09-18 | E | 5 | Mucinous cystadenocarcinoma, NOS | WELL DIFFERENTIATED | X |  | / |  |
| 09-18 | E | 6 | Clear cell adenocarcinoma, NOS | UNDIFFERENTIATED | X |  | 1 | 60% |
| 09-18 | E | 7 | Clear cell adenocarcinoma, NOS | UNDIFFERENTIATED | X |  | 1 | 60% |
| 09-18 | E | 8 | Mucinous cystadenocarcinoma, NOS | WELL DIFFERENTIATED | 1A |  | 3 | 70% |
| 09-18 | E | 9 | Mucinous cystadenocarcinoma, NOS | WELL DIFFERENTIATED | 1A |  | 3 | 70% |
| 09-18 | E | 10 | Endometrioid adenocarcinoma, NOS | WELL DIFFERENTIATED | 1A |  | 2 | 60% |
| 09-18 | E | 11 | Endometrioid adenocarcinoma, NOS | WELL DIFFERENTIATED | 1A |  | 1 | 60% |
| 09-18 | E | 12 | Endometrioid adenocarcinoma, NOS | POORLY DIFFERENTIATED | 1C |  | / |  |
| 09-18 | E | 13 | Endometrioid adenocarcinoma, NOS | POORLY DIFFERENTIATED | 1C |  | / |  |
| 09-18 | F | 2 | Endometrioid adenocarcinoma, NOS | UNDIFFERENTIATED | 1C |  | 2 | 90% |
| 09-18 | F | 3 | Endometrioid adenocarcinoma, NOS | UNDIFFERENTIATED | 1C |  | 2 | 40% |
| 09-18 | F | 4 | Clear cell adenocarcinoma, NOS | MODERATELY DIFFERENTIATED | 1C |  | 1 | 90% |
| 09-18 | F | 5 | Clear cell adenocarcinoma, NOS | MODERATELY DIFFERENTIATED | 1C |  | 1 | 90% |
| 09-18 | F | 6 | Clear cell adenocarcinoma, NOS | UNDIFFERENTIATED | X | 2 | 0 |  |
| 09-18 | F | 7 | Clear cell adenocarcinoma, NOS | UNDIFFERENTIATED | X | 2 | 0 |  |
| 09-18 | F | 8 | Endometrioid adenocarcinoma, NOS | POORLY DIFFERENTIATED | 2C |  | 2 | 10% |
| 09-18 | F | 9 | Endometrioid adenocarcinoma, NOS | POORLY DIFFERENTIATED | 2C |  | 2 | 5% |
| 09-18 | F | 10 | Clear cell adenocarcinoma, NOS | NOT DETERMINED; N/A; UNK PRIMARIES, ETC. | 1C |  | 0 |  |
| 09-18 | F | 11 | Clear cell adenocarcinoma, NOS | NOT DETERMINED; N/A; UNK PRIMARIES, ETC. | 1C |  | 0 |  |
| 09-18 | F | 12 | Serous surface papillary carcinoma | UNDIFFERENTIATED | 3C |  | / |  |
| 09-18 | F | 13 | Serous surface papillary carcinoma | UNDIFFERENTIATED | 3C |  | / |  |
| 09-18 | G | 2 | Clear cell adenocarcinoma, NOS | NOT DETERMINED; N/A; UNK PRIMARIES, ETC. | 2C |  | 1 | 60% |
| 09-18 | G | 3 | Clear cell adenocarcinoma, NOS | NOT DETERMINED; N/A; UNK PRIMARIES, ETC. | 2C |  | / |  |
| 09-18 | G | 4 | Mucinous cystadenocarcinoma, NOS | NOT DETERMINED; N/A; UNK PRIMARIES, ETC. | 1C |  | 2 | 80% |
| 09-18 | G | 5 | Mucinous cystadenocarcinoma, NOS | NOT DETERMINED; N/A; UNK PRIMARIES, ETC. | 1C |  | 2 | 80% |
| 09-18 | G | 6 | Mucinous cystadenocarcinoma, NOS | WELL DIFFERENTIATED | X |  | / |  |
| 09-18 | G | 7 | Mucinous cystadenocarcinoma, NOS | WELL DIFFERENTIATED | X |  | / |  |
| 09-18 | G | 8 |  |  |  |  |  |  |
| 09-18 | G | 9 |  |  |  |  |  |  |
| 09-18 | G | 10 |  |  |  |  |  |  |
| 09-18 | G | 11 |  |  |  |  |  |  |
| 09-18 | G | 12 |  |  |  |  |  |  |
| 09-18 | G | 13 |  |  |  |  |  |  |
| 09-19 | C | 2 | Adenocarcinoma, NOS | POORLY DIFFERENTIATED | X |  | 3 | 90% |
| 09-19 | C | 3 | Adenocarcinoma, NOS | POORLY DIFFERENTIATED | X |  | 2 | 80% |
| 09-19 | C | 4 | Adenocarcinoma, NOS | POORLY DIFFERENTIATED | X |  | 1 | 80% |
| 09-19 | C | 5 | Adenocarcinoma, NOS | POORLY DIFFERENTIATED | X |  | 1 | 70% |
| 09-19 | C | 6 | Serous surface papillary carcinoma | POORLY DIFFERENTIATED | 1A |  | 2 | 30% |
| 09-19 | C | 7 | Serous surface papillary carcinoma | POORLY DIFFERENTIATED | 1A |  | 2 | 10% |
| 09-19 | C | 8 | Serous surface papillary carcinoma | POORLY DIFFERENTIATED | 1A |  | 2 | 60% |
| 09-19 | C | 9 | Serous surface papillary carcinoma | POORLY DIFFERENTIATED | 1A |  | 2 | 60% |
| 09-19 | C | 10 | Papillary serous cystadenocarcinoma | UNDIFFERENTIATED | X |  | 1 | 50% |
| 09-19 | C | 11 | high-grade serous carcinoma | UNDIFFERENTIATED | X |  | 1 | 50% |
| 09-19 | C | 12 | high-grade serous carcinoma | UNDIFFERENTIATED | X |  | 1 | 40% |
| 09-19 | C | 13 | high-grade serous carcinoma | UNDIFFERENTIATED | X |  | 1 | 40% |
| 09-19 | D | 2 | Serous surface papillary carcinoma | NOT DETERMINED; N/A; UNK PRIMARIES, ETC. | X |  | / |  |
| 09-19 | D | 3 | Serous surface papillary carcinoma | NOT DETERMINED; N/A; UNK PRIMARIES, ETC. | X |  | 2 | 90% |
| 09-19 | D | 4 | high-grade serous carcinoma | NOT DETERMINED; N/A; UNK PRIMARIES, ETC. | X |  | 0 |  |
| 09-19 | D | 5 | high-grade serous carcinoma | NOT DETERMINED; N/A; UNK PRIMARIES, ETC. | X |  | 1 | 20% |
| 09-19 | D | 6 | high-grade serous carcinoma | NOT DETERMINED; N/A; UNK PRIMARIES, ETC. | 3C |  | 2 | 20% |
| 09-19 | D | 7 | high-grade serous carcinoma | NOT DETERMINED; N/A; UNK PRIMARIES, ETC. | 3C |  | 1 | 20% |
| 09-19 | D | 8 | Papillary serous cystadenocarcinoma | NOT DETERMINED; N/A; UNK PRIMARIES, ETC. | 3C |  | 3 | 80% |
| 09-19 | D | 9 | high-grade serous carcinoma | NOT DETERMINED; N/A; UNK PRIMARIES, ETC. | 3C |  | 3 | 80% |
| 09-19 | D | 10 | Serous surface papillary carcinoma | WELL DIFFERENTIATED | X |  | 1 | 20% |
| 09-19 | D | 11 | Serous surface papillary carcinoma | WELL DIFFERENTIATED | X |  | 1 | 20% |
| 09-19 | D | 12 | high-grade serous carcinoma | POORLY DIFFERENTIATED | 3C |  | 2 | 60% |
| 09-19 | D | 13 | high-grade serous carcinoma | POORLY DIFFERENTIATED | 3C |  | / |  |
| 09-19 | E | 2 | Serous surface papillary carcinoma | NOT DETERMINED; N/A; UNK PRIMARIES, ETC. | X |  | 2 | 70% |
| 09-19 | E | 3 | Serous surface papillary carcinoma | NOT DETERMINED; N/A; UNK PRIMARIES, ETC. | X |  | 2 | 40% |
| 09-19 | E | 4 | high-grade serous carcinoma | MODERATELY DIFFERENTIATED | 3C |  | 2 | 20% |
| 09-19 | E | 5 | high-grade serous carcinoma | MODERATELY DIFFERENTIATED | 3C |  | 2 | 20% |
| 09-19 | E | 6 | Serous cystadenocarcinoma, NOS | POORLY DIFFERENTIATED | 3C |  | 1 | 70% |
| 09-19 | E | 7 | high-grade serous carcinomaNOS | POORLY DIFFERENTIATED | 3C |  | 1 | 40% |
| 09-19 | E | 8 | high-grade serous carcinoma | UNDIFFERENTIATED | 3C |  | 1 | 10% |
| 09-19 | E | 9 | high-grade serous carcinoma | UNDIFFERENTIATED | 3C |  | 1 | 30% |
| 09-19 | E | 10 | high-grade serous carcinoma | POORLY DIFFERENTIATED | 1C |  | / |  |
| 09-19 | E | 11 | high-grade serous carcinoma | POORLY DIFFERENTIATED | 1C |  | 1 | 40% |
| 09-19 | E | 12 | high-grade serous carcinoma | POORLY DIFFERENTIATED | X | 3 | 0 |  |
| 09-19 | E | 13 | high-grade serous carcinoma | POORLY DIFFERENTIATED | X | 3 | 0 |  |
| 09-19 | F | 2 | high-grade serous carcinoma | POORLY DIFFERENTIATED | X |  | 1 | 40% |
| 09-19 | F | 3 | high-grade serous carcinoma | POORLY DIFFERENTIATED | X |  | 1 | 60% |
| 09-19 | F | 4 | high-grade serous carcinoma | UNDIFFERENTIATED | 1C |  | 0 |  |
| 09-19 | F | 5 | high-grade serous carcinoma | UNDIFFERENTIATED | 1C |  | 0 |  |
| 09-19 | F | 6 | Adenocarcinoma, NOS | NOT DETERMINED; N/A; UNK PRIMARIES, ETC. | X |  | 1 | 40% |
| 09-19 | F | 7 | Adenocarcinoma, NOS | NOT DETERMINED; N/A; UNK PRIMARIES, ETC. | X |  | 1 | 40% |
| 09-19 | F | 8 | high-grade serous carcinoma | WELL DIFFERENTIATED | X |  | / |  |
| 09-19 | F | 9 | high-grade serous carcinoma | WELL DIFFERENTIATED | X |  | 2 | 80% |
| 09-19 | F | 10 | Serous surface papillary carcinoma | MODERATELY DIFFERENTIATED | 3C |  | 0 |  |
| 09-19 | F | 11 | Serous surface papillary carcinoma | MODERATELY DIFFERENTIATED | 3C |  | 0 |  |
| 09-19 | F | 12 | Serous surface papillary carcinoma | MODERATELY DIFFERENTIATED | X |  | 2 | 70% |
| 09-19 | F | 13 | Serous surface papillary carcinoma | MODERATELY DIFFERENTIATED | X |  | 2 | 70% |
| 09-19 | G | 2 | high-grade serous carcinoma | UNDIFFERENTIATED | 3C |  | 2 | 90% |
| 09-19 | G | 3 | high-grade serous carcinoma | UNDIFFERENTIATED | 3C |  | 2 | 90% |
| 09-19 | G | 4 | high-grade serous carcinoma | POORLY DIFFERENTIATED | X |  | 0 |  |
| 09-19 | G | 5 | high-grade serous carcinoma | POORLY DIFFERENTIATED | X |  | 0 |  |
| 09-19 | G | 6 | high-grade serous carcinoma | POORLY DIFFERENTIATED | 3C |  | 2 | 80% |
| 09-19 | G | 7 | high-grade serous carcinoma | POORLY DIFFERENTIATED | 3C |  | 2 | 80% |
| 09-19 | G | 8 | high-grade serous carcinoma | POORLY DIFFERENTIATED | X |  | 0 |  |
| 09-19 | G | 9 | high-grade serous carcinoma | POORLY DIFFERENTIATED | X |  | 0 |  |
| 09-19 | G | 10 | high-grade serous carcinoma | POORLY DIFFERENTIATED | X |  | / |  |
| 09-19 | G | 11 | high-grade serous carcinoma | POORLY DIFFERENTIATED | X |  | / |  |
| 09-19 | G | 12 | Normal ovarian and tubal epithelia |  |  | 3 |  |  |
| 09-19 | G | 13 | Normal ovarian and tubal epithelia |  |  | 3 |  |  |

**Note:** The ovarian carcinoma TMA contains two cores per tumor tissues. A series of antibody dilutions were tested, and the slides stained using the most appropriate concentration of the anti-lamin A/C antibodies were used for reading. The staining intensity was estimated and scored as 3, strongly positive; 2, positive; 1, weakly positive; and 0, absent. The percentages of tumor cells positive for staining were also estimated.

**Abbreviation:** “/”, no information because that the tissue core is defective or no tumor or epithelial cells present; NOE, normal ovarian epithelia; NOS, Not Otherwise Specified; UNK, unknown; “X”, no information.
